# Metrics of sexual behavior stigma among cisgender men who have sex with men in Mexico: exploratory and confirmatory factor analyses

**DOI:** 10.1186/s12879-022-07672-0

**Published:** 2022-08-13

**Authors:** John Mark Wiginton, Sarah M. Murray, Angel B. Algarin, Stefan D. Baral, Travis H. Sanchez, Laramie R. Smith

**Affiliations:** 1 School of Social Work, San Diego State University, 5500 Campanile Drive, San Diego, CA 92182 USA; 2 Department of Medicine, University of California-San Diego, San Diego, USA; 3 Department of Mental Health, Johns Hopkins University Bloomberg School of Public Health, Baltimore, MD USA; 4 Division of Infectious Diseases and Global Public Health, University of California San Diego, CA San Diego, USA; 5 Center for Public Health & Human Rights, Department of Epidemiology, Johns Hopkins University Bloomberg School of Public Health, Baltimore, MD USA; 6 Department of Epidemiology, Emory University Rollins School of Public Health, Atlanta, GA USA

**Keywords:** Men who have sex with men, Sexual behavior stigma, Mexico, Factor analysis, Construct validity

## Abstract

Cisgender gay, bisexual, and other men who have sex with men (MSM) in Mexico experience disparities in sexual health outcomes, perhaps most notably in HIV prevalence, HIV testing and status awareness, and condom use. Sexual behavior stigma, underpinned by socio-structural factors specific to Mexico (e.g., machismo), uniquely shapes these sexual health disparities. However, few reliable, valid measures are available to document, track, and ultimately mitigate sexual behavior stigma in this context. Exploratory and confirmatory factor analyses were performed on responses to a 13-item sexual behavior stigma scale from 15,681 MSM recruited online across Mexico. Associations with extracted factors were tested to assess construct validity. Three subscales were identified in exploratory factor analysis and validated in confirmatory factor analysis: “stigma from family and friends” (α = 0.65), “anticipated healthcare stigma” (α = 0.84), and “general social stigma” (α = 0.70). External construct validity was indicated through each subscale’s strong association (all p < 0.001) with perceived community intolerance of MSM and perceived community discrimination toward people living with HIV. These subscales show promise as reliable, valid measures for assessing sexual behavior stigma among MSM in Mexico, and as tools for documenting and tracking sexual behavior stigma trends, comparing regional burdens of sexual behavior stigma, and tracking the progress of stigma-mitigation interventions among MSM in Mexico. Future research is needed to understand the extent to which each subscale is differentially associated with sexual (and other) health outcomes, which can inform the development and implementation of uniquely tailored stigma-mitigation, HIV-prevention, HIV-care, and other needed interventions for MSM in Mexico.

**Supplementary Information:**

The online version contains supplementary material available at 10.1186/s12879-022-07672-0.

## Introduction

Globally, cisgender gay, bisexual, and other men who have sex with men (MSM) are often stigmatized for engaging in same-sex practices [1, 2]. Such sexual behavior stigma, which can be perceived, enacted, anticipated, or internalized [3–5], has been widely reported across diverse world regions and countries of different resource levels [6–8]. Though Latin American contexts are considered to be among the more inclusive and protective regions for sexual minorities [7], MSM in Latin America continue to report discrimination, violence, and other forms of sexual behavior stigma that affect their quality of life and hamper HIV prevention efforts [9, 10].

In Mexico in particular, sexual behavior stigma continues to permeate communities and society at large [11–17], despite recent human rights and legislative progress for sexual minorities [18, 19]. In a recent national survey, two thirds of respondents believed the rights of sexual minorities were not respected in Mexican society, and two thirds believed there was little or no justification for two people of the same sex to live as a couple [16]. Socio-cultural factors common to the Mexican context may in part underlie and shape how sexual behavior stigma and stigma-linked outcomes manifest and are experienced by MSM. Among the most notable of these factors are *machismo* (one’s masculinity/manhood must be proven through power, dominance, risk-taking, sexual prowess, low sexual control, self-sufficiency, non-effeminacy), *familismo* (prioritization of the family over oneself and of one’s family’s needs and wishes over one’s own), fatalism (one’s circumstances, including health, are unavoidable and controlled by external forces such as fate or god), and Catholic influence on same-sex sexuality views [20–22].

MSM in Mexico experience a disproportionate burden of HIV, with an estimated prevalence of 12–17% [23–25] compared to < 0.3% in the general population [26]. HIV testing and status awareness remain low at 40%, while roughly 1 in 3 do not use condoms [25]. Consistent with findings from other regions [27–33], sexual behavior stigma has been linked to risk factors for HIV infection and transmission among MSM in Mexico, including sexual risk behavior [11], low uptake of HIV prevention tools [34, 35], poor HIV care and treatment outcomes [11, 36], and psychosocial risk factors for HIV [11, 37, 38]. Sexual behavior stigma may also underlie infrequent/delayed HIV testing, late HIV diagnosis, and suboptimal linkage to HIV care and treatment services for MSM in Mexico, resulting in late initiation of universally-available antiretroviral treatment (ART) [39–41].

Given existing evidence of associations between sexual behavior stigma and HIV outcomes in Mexico, more research is critically needed to understand how stigma may impact the health of MSM and track the effectiveness of HIV prevention and response efforts. However, the rigor and success of research on these topics requires accurate and nuanced measures of sexual behavior stigma [15, 16]. Existing reviews of the broader literature reveal inconsistent use of extant stigma measures and a general lack of reliable and valid sexuality-based stigma measures for use in in low-resource settings specifically; further, sexual behavior stigma scales are often narrowly-focused on only one manifestation of stigma (e.g., internalized or enacted stigma only) [8, 42, 43].

Assessing multiple manifestations of sexual behavior stigma is crucial to capturing a broader, fuller range of salient stigma domains and can provide a more nuanced picture of MSM’s lived experience as sexual minorities. Moreover, assorted forms of stigma have been shown to be differentially associated with HIV outcomes and related vulnerabilities among MSM in diverse contexts worldwide [44–47]. Among MSM in Mexico specifically, enacted stigma has been associated with limited HIV care access and decreased ART adherence, psychological distress, and sexual violence [11, 36–38]; internalized stigma has been associated with low uptake of HIV testing [34]; and perceived/anticipated stigma has been associated with decreased uptake of HIV pre-exposure prophylaxis (PrEP) and constrained access to sexual health knowledge [11, 35]. These findings illustrate the need for comprehensive measures that distinguish between and separately assess diverse stigma manifestations.

In Mexico, stigma among MSM has been explored qualitatively [11, 13] or been restricted to one type of stigma, such as internalized (e.g., assessed with the Internalized Homophobia scale) [34, 37, 48–51] or enacted stigma (e.g., via Experiences of Homophobia or other scales) [36–38, 48, 52], with stigma often being an incidental rather than primary focus of inquiry. Moreover, there is minimal evidence that any sexuality-based stigma scale has been thoroughly validated among MSM in Mexico. To fill these gaps, we sought to explore the psychometrics and construct validity of a scale that measures multiple manifestations of sexual behavior stigma (perceived, anticipated, enacted) in a sample of Mexican MSM that has previously shown stability in structure, high internal consistency, and utility across multiple low-resource settings in sub-Saharan Africa [53] and samples of MSM across the United States (US) [53, 54]. Specifically, we aimed to assess and validate the factor structure of the stigma scale, assess the reliability of the factors underlying the scale, and assess the external construct validity of the scale using data from a nationwide sample of MSM in Mexico.

We hypothesized a three-factor structure, given findings with MSM in other regions (US, sub-Saharan Africa) with this scale [53, 54]. We also hypothesized that MSM who perceived less tolerance of sexual minorities in their immediate community would also be likely to have encountered experiences of sexual behavior stigma themselves, as prevalent societal stigma toward MSM has been documented in Mexico [15, 16]. Likewise, as HIV-related stigma and sexual behavior stigma among MSM have long been linked in numerous contexts [55–58], including Mexico [59], we hypothesized that MSM who perceived community discrimination toward people living with HIV in their immediate community would also be likely to have encountered experiences of sexual behavior stigma themselves. Finally, we hypothesized that associations between different manifestations of sexual behavior stigma and community discrimination toward people living with HIV would be stronger than those of stigma with community intolerance of sexual minorities, on account of overlapping HIV and sexual behavior stigma. In other words, sexual behavior stigma may intersect with HIV-stigma such that MSM’s personal experiences of stigma are intensified in communities that are perceived as stigmatizing toward people living with HIV (PLHIV).

## Methods

### Data source, sampling procedures, and participants

Data come from Encuesta de Sexo Entre Hombres (ES Entre Hombres), a collaborative study conducted by researchers from Mexico's National Institute of Public Health, Emory University, and University of California-San Diego. Detailed methods for ES Entre Hombres have been published elsewhere [60]. Briefly, non-probability-based sampling was used to recruit participants online using advertisements on social media (e.g., Facebook), webpages catering to men who have sex with men (e.g., SoyHomosensual), and smartphone dating or hookup applications (e.g., Grindr) between May and July 2017 across Mexico’s six geographic zones: Northwest (Baja California, Baja California Sur, Sonora, Sinaloa, Chihuahua, Durango); Northeast (Coahuila, Nuevo León, Tamaulipas, San Luis Potosí, Zacatecas); Bajío/Occidente (Aguascalientes, Nayarit, Jalisco, Colima, Guanajuato, Michoacán, Querétaro); Central (Hidalgo, Puebla, Tlaxcala, Morelos, Guerrero, Veracruz); CDMX/EdoMex (State of Mexico, Mexico City); and South/Southeast (Oaxaca, Tabasco, Chiapas, Campeche, Yucatán, Quintana Roo).

Eligibility criteria included being cisgender male; age ≥ 18 years; report of lifetime oral or anal sex with another man or identification as homosexual/gay or bisexual; and residence in the aforementioned geographic zones. After providing informed consent, participants immediately began the online survey, which was hosted by SurveyGizmo [61] and informed primarily by the American Men’s Internet Survey [62, 63], followed by the Survey of Seroprevalence in MSM Encounter Sites in Mexico [64] and personnel from Mexico’s National Center for the Prevention and Control of HIV and AIDS. The American Men’s Internet Survey (which included the sexual behavior stigma items) was translated from English to Spanish via a translation service (ISO 9001, ISO 17100 and EN 15038-compliant) (Dynamic Language), after which two bilingual (Spanish and English) colleagues reviewed the translation for accuracy and appropriateness. A third bilingual colleague who resides in Mexico and is a native Spanish speaker with expertise in HIV among MSM in Mexico reviewed the survey in its final form to ensure its suitability for the target sample. In addition to sexual behavior stigma, the survey assessed HIV testing and status; sexual and drug use behaviors; experiences with medical care and treatment for people living with HIV; and the use of HIV-prevention services. The study was approved by the ethics committee of Mexico's National Institute of Public Health and the institutional review boards of Emory University and University of California-San Diego.

### Measures

#### Sociodemographic and other variables

We ascertained several sociodemographic characteristics (age, education, employment status, sexual identity, relationship status, living situation, region), HIV status, and whether or not participants had disclosed their same-sex attraction or behaviors to healthcare providers; lesbian, gay, bisexual, transgender [LGBT] friends; heterosexual friends; family members; employers/teachers; and classmates/colleagues.

#### Sexual behavior stigma items

Participants responded to 13 sexual behavior stigma items (Table 1; Spanish version presented to participants in Additional file 1: Appendix) that were previously developed using a socioecological framework through research with MSM in sub-Saharan Africa and have since been used with MSM in the US [4, 53, 54]. More details on item and scale-development have been published elsewhere [53], but in brief, items assessed lifetime experiences of perceived, anticipated, and enacted sexual behavior stigma in social, healthcare, and community contexts. Response options for each item included “Yes,” “No,” “Not applicable,” “Prefer not to answer,” and “I don’t know.” Only “yes” and “no” responses were considered for analysis, with others treated as missing. Enacted stigma in the form of sexual violence (item 13) was assessed in two parts: (a) experience (whether the participant had experienced sexual violence) and (b) attribution (whether they believed the sexual violence was related to their having sex with men). Endorsement of both the experience of sexual violence and the belief that it was related to having sex with men was coded as an affirmative response, while no experience or experience without attribution to sexual behavior was coded as “No.” Item 12 (physical violence) was intended to be assessed in the same manner. However, during exploratory data analysis it was discovered that the attribution portion had been inadvertently included in both parts of the item. In other words, the first part of the item assessed both experience and attribution (whether they had experienced physical violence that was related to their having sex with men), and the second part assessed attribution again. We therefore considered responses from the first portion of the question only.

**Table 1:** Endorsement of sexual behavior stigma items administered to cisgender men who have sex with men in Mexico, 2017

Item	Description (stigma type)	Response options	EFA sample (n = 7841) n (%)	CFA sample (n = 7840) n (%)	Total sample (N = 15,681) n (%)
1	Have you ever felt excluded from family activities because you have sex with men? (perceived)	Yes	1666 (21.2)	1634 (20.8)	3300 (21.0)
		No	5373 (68.5)	5447 (69.5)	10,820 (69.0)
		Unknown	802 (10.2)	759 (9.7)	1561 (10.0)
2	Have you ever felt that family members have made discriminatory remarks or gossiped about you because you have sex with men? (perceived)	Yes	3190 (40.7)	3260 (41.6)	6450 (41.1)
		No	3848 (49.1)	3806 (48.5)	7654 (48.8)
		Unknown	803 (10.2)	774 (9.9)	1577 (10.1)
3	Have you ever felt rejected by your friends because you have sex with men? (perceived)	Yes	1515 (19.3)	1536 (19.6)	3051 (19.5)
		No	5896 (75.2)	5860 (74.7)	11,756 (75.0)
		Unknown	430 (5.5)	444 (5.7)	874 (5.6)
4	Have you ever felt afraid to go to healthcare services because you worry someone may learn you have sex with men? (anticipated)	Yes	1733 (22.1)	1777 (22.7)	3510 (22.4)
		No	5873 (74.9)	5813 (74.1)	11,686 (74.5)
		Unknown	235 (3.0)	250 (3.2)	485 (3.1)
5	Have you ever avoided going to healthcare services because you worry someone may learn you have sex with men? (anticipated)	Yes	1226 (15.6)	1241 (15.8)	2467 (15.7)
		No	6401 (81.6)	6378 (81.4)	12,779 (81.5)
		Unknown	214 (2.7)	221 (2.8)	435 (2.8)
6	Have you ever felt that you were not treated well in a health center because someone knew that you have sex with men? (perceived)	Yes	650 (8.3)	643 (8.2)	1293 (8.2)
		No	6542 (83.4)	6546 (83.5)	13,088 (83.5)
		Unknown	649 (8.3)	651 (8.3)	1300 (8.3)
7	Have you ever heard healthcare providers gossiping about you (talking about you) because you have sex with men? (enacted)	Yes	622 (7.9)	595 (7.6)	1217 (7.8)
		No	6621 (84.4)	6616 (84.4)	13,237 (84.4)
		Unknown	598 (7.6)	629 (8.0)	1227 (7.8)
8	Have you ever felt that the police refused to protect you because you have sex with men? (perceived)	Yes	1151 (14.7)	1153 (14.7)	2304 (14.7)
		No	5649 (72.0)	5620 (71.7)	11,269 (71.9)
		Unknown	1041 (13.3)	1067 (13.6)	2108 (13.4)
9	Have you ever felt scared to be in public places because you have sex with men? (anticipated)	Yes	1888 (24.1)	1868 (23.8)	3756 (24.0)
		No	5576 (71.1)	5582 (71.2)	11,158 (71.2)
		Unknown	377 (4.8)	390 (5.0)	767 (4.9)
10	Have you ever been verbally harassed and felt it was because you have sex with men? (enacted)	Yes	3670 (46.8)	3585 (45.7)	7255 (46.3)
		No	3867 (49.3)	3938 (50.2)	7805 (49.8)
		Unknown	304 (3.9)	317 (4.0)	621 (4.0)
11	Have you ever been blackmailed by someone because you have sex with men? (enacted)	Yes	1352 (17.2)	1313 (16.7)	2665 (17.0)
		No	6273 (80.0)	6305 (80.4)	12,578 (80.2)
		Unknown	216 (2.8)	222 (2.8)	438 (2.8)
12	Has someone ever physically hurt you (pushed, shoved, slapped, hit, kicked, choked or otherwise physically hurt you)? [AND] Do you believe any of these experiences of physical violence was/were related to the fact that you have sex with men? (enacted)	Yes	1249 (15.9)	1238 (15.8)	2487 (15.9)
		No	6356 (81.1)	6359 (81.1)	12,715 (81.1)
		Unknown	236 (3.0)	243 (3.1)	479 (3.1)
13	Have you ever been forced to have sex when you did not want to (by forced, I mean physically forced, coerced to have sex, or penetrated with an object, when you did not want to)? [AND] Do you believe any of these experiences of sexual violence were related to the fact that you have sex with men? (enacted)	Yes	534 (6.8)	546 (7.0)	1080 (6.9)
		No	6913 (88.2)	6901 (88.0)	13,814 (88.1)
		Unknown	394 (5.0)	393 (5.0)	787 (5.0)

EFA, exploratory factor analysis; CFA, confirmatory factor analysis

#### External construct validity items

Two items adapted from the Centers for Disease Control and Prevention’s National HIV Behavioral Surveillance survey assessed external construct validity. One item assessed perceived community tolerance of sexual minorities (“Most of the people in my area are tolerant of homosexuals and bisexuals”), and another item assessed perceived community discrimination toward people living with HIV (“Most people in my area would discriminate against someone who has HIV”) [65]. Participants responded to each item on a five-point Likert scale, ranging from “Strongly agree” (1) to “Strongly disagree” (5) (additional response options of “Prefer not to answer” and “I don’t know” were treated as missing).

### Analyses

All analyses were conducted in Stata Version 15 [66] and Mplus Version 8 [67]. Deduplicated surveys from eligible, consenting participants that were at least 70% complete were analyzed. Item-level missingness was assessed for the stigma scale, and descriptive statistics were calculated for sociodemographic characteristics. We then randomly split the sample into two subsamples of relatively equal sizes. Descriptive statistics were calculated separately for stigma items in the two subsamples and compared.

#### Exploratory factor analysis

In the first random subsampled dataset, we performed an exploratory factor analysis (EFA). As a preliminary step, we calculated the Kaiser–Meyer–Olkin (KMO) test of sampling adequacy, which measures the proportion of variance in variables (e.g., stigma items) that may be driven by underlying factors. A KMO ≥ 0.50 indicates adequate sampling to detect underlying factors and data suitability for factor analysis [68]. A principal components analysis was then conducted on a tetrachoric correlation matrix given that all included items were dichotomous. Next, a scree plot was generated, and a parallel analysis was performed. The number of eigenvalues > 1, scree plot, results of the parallel analysis, and scientific interpretation of the resulting factors were considered in determining the number of factors to specify in EFA. The EFA was conducted with robust weighted least squares estimation, given its tendency to encounter fewer convergence problems than other methods [67, 69]. A quartimin rotation was used based on an expectation that factors would be correlated and due to its potential to minimize item complexity (i.e., cross-loadings) and yield a more simplified factor structure [53, 54, 70–73]. Resulting item loadings were examined with attention placed on low-loading items (< 0.40), cross-loading items (i.e., those that loaded ≥ 0.40 on a main factor but also ≥ 0.30 on a second factor, with a difference of ≤ 0.20 between the two loadings), and overall factor interpretability [74]. Any low- or cross-loading items were removed one-by-one, and EFA procedures were repeated until such issues were resolved. The following statistics were used to assess adequate model fit: Root Mean Square Error of Approximation (RMSEA) < 0.05; Comparative Fit Index (CFI) > 0.90; Tucker Lewis Index (TLI) > 0.90; and, Standardized Root Mean Square Residual (SRMR) < 0.08 [75–78]. Final EFA model selection was based on the number of strongly-loading items per factor, interpretability, parsimony, and fit indices. Cronbach’s alpha (≥ 0.70 considered adequate) was calculated to assess the internal consistency of each factor’s items [79].

#### Confirmatory factor analysis

Confirmatory factor analysis (CFA) was performed on the second subsample to establish factorial validity, i.e., to validate the relationships between factors and items and the relationships between factors themselves that emerged in the EFA. The same fit statistics described above were used to assess model goodness of fit.

#### External construct validity

To assess external construct validity, we used a structural equation modeling approach by modeling the community intolerance and community discrimination items as structural parameters in the CFA model.

#### Sensitivity analyses

An initial sensitivity analysis was performed in which all EFA and CFA procedures described above were repeated using a complete-case approach. An additional sensitivity analysis was performed using an oblique equamax rotation criterion to depict potential item complexities that may have been minimized or masked when using the quartimin rotation criterion [72, 73]. Lastly, the two approaches were combined to conduct a complete-case analysis using an equamax rotation criterion.

## Results

### Sample characteristics

**Fig. 1:**
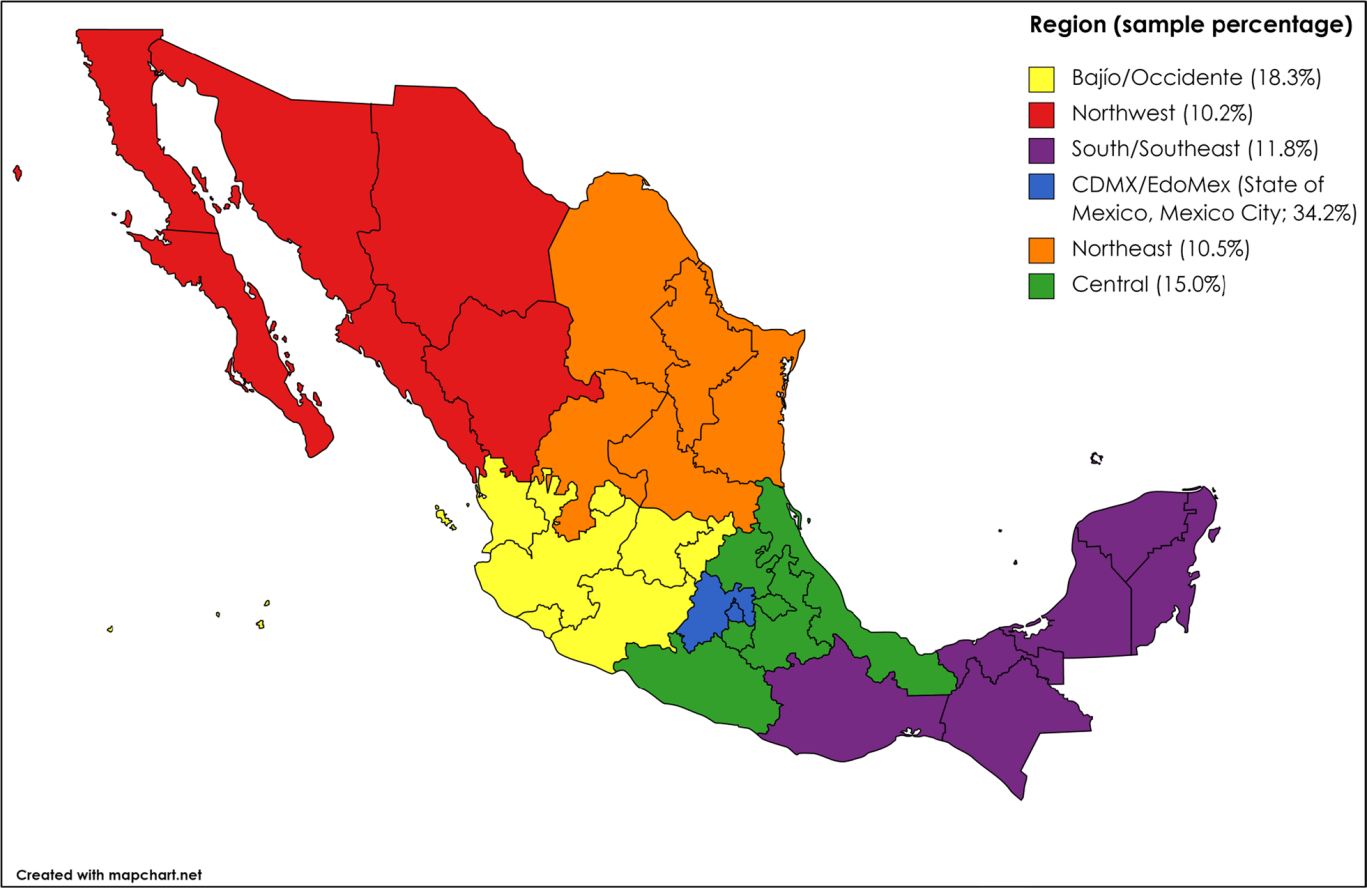
Representation of Mexico’s six geographic zones by Encuesta de Sexo Entre Hombres participants, 2017 (N = 15,681)

A total of 15,875 participants completed the survey; 194 did not complete any of the stigma items and were excluded, leaving data from 15,681 participants in the analytic sample (EFA subsample: n = 7841; CFA subsample: n = 7840). In the combined analytic sample, mean age was 28.1 years, and median age was 26 years, with roughly two thirds of participants < 30 years. Just over half had earned a bachelor’s degree, and just under half were employed. Almost half lived with family, and 30% were in a stable relationship. Roughly one in ten were living with HIV. More than 80% identified as gay and had disclosed their same-sex attraction or practices to LGBT friends, with roughly half having disclosed to a healthcare provider. More than a third resided in CDMX/EdoMex (Mexico City/State of Mexico; Fig. 1). Characteristics were comparable between EFA and CFA subsamples (Table 2).

**Table 2:** Sociodemographic characteristics and perceived community stigma among cisgender men who have sex with men in Mexico, 2017

	EFA sample (n = 7841)	CFA sample (n = 7840)	Total (N = 15,681)
Sociodemographic characteristics			
Age, mean (SD); median (IQR) χ^2^ (p-value)	28.0 (8.2); 26 (22–32) –	28.2 (8.1); 26 (22–32) 4.23 (0.04)	28.1 (8.1); 26 (22–32) –
Age categories, n (%)			
18–24	3164 (40.4)	3041 (38.8)	6205 (39.6)
25–29	2022 (25.8)	2097 (26.7)	4119 (26.3)
30–34	1233 (15.7)	1249 (15.9)	2482 (15.8)
35–39	648 (8.3)	721 (9.2)	1369 (8.7)
40+	774 (9.9)	732 (9.3)	1506 (9.6)
χ^2^ (p-value)	–	8.97 (0.06)	–
Education			
Secondary or less	200 (2.6)	192 (2.4)	392 (2.5)
High school	1787 (22.8)	1716 (21.9)	3503 (22.3)
Technical	789 (10.1)	732 (9.3)	1521 (9.7)
Bachelor’s	3959 (50.5)	4051 (51.7)	8010 (51.1)
Postgraduate	986 (12.6)	1001 (12.8)	1987 (12.7)
Missing/unknown	120 (1.5)	148 (1.9)	268 (1.7)
χ^2^ (p-value)	–	4.85 (0.30)	–
Employment status			
Unemployed	484 (6.2)	501 (6.4)	985 (6.3)
Employed	3503 (44.7)	3557 (45.4)	7060 (45.0)
Student	1486 (19.0)	1374 (17.5)	2860 (18.2)
Employed student	1383 (17.6)	1414 (18.0)	2797 (17.8)
Has own business	651 (8.3)	667 (8.5)	1318 (8.4)
Other	58 (0.7)	53 (0.7)	111 (0.7)
Missing/unknown	276 (3.5)	274 (3.5)	550 (3.5)
χ^2^ (p-value)	–	5.86 (0.32)	–
Relationship status			
In a stable relationship	2370 (30.2)	2401 (30.6)	4771 (30.4)
Not in a stable relationship	5103 (65.1)	5081 (64.8)	10,184 (64.9)
Missing/Unknown	368 (4.7)	358 (4.6)	726 (4.6)
χ^2^ (p-value)	–	0.24 (0.62)	–
Living situation			
With family	3869 (49.3)	3738 (47.7)	7607 (48.5)
With others or alone	2368 (30.2)	2380 (30.4)	4748 (30.3)
Missing/unknown	1604 (20.5)	1722 (22.0)	3326 (21.2)
χ^2^ (p-value)	–	6.47 (0.04)	–
HIV status			
Negative	4360 (55.6)	4368 (55.7)	8728 (55.7)
Positive	818 (10.4)	820 (10.5)	1638 (10.4)
Unknown	2663 (34.0)	2652 (33.8)	5315 (33.9)
χ^2^ (p-value)	–	3.97 (0.55)	–
Sexual identity			
Gay	6353 (81.0)	6428 (82.0)	12,781 (81.5)
Bisexual	1348 (17.2)	1277 (16.3)	2625 (16.7)
Heterosexual	53 (0.7)	43 (0.5)	96 (0.6)
Questioning	45 (0.6)	57 (0.7)	102 (0.7)
Missing/unknown	42 (0.5)	35 (0.4)	77 (0.5)
χ^2^ (p-value)	–	4.81 (0.19)	–
Sexuality disclosure to a healthcare provider			
Yes	3921 (50.0)	3927 (50.1)	7848 (50.0)
No	3920 (50.0)	3913 (49.9)	7833 (50.0)
χ^2^ (p-value)	–	0.04 (0.84)	–
Sexuality disclosure to LGBT friends			
Yes	6434 (82.1)	6396 (81.6)	12,830 (81.8)
No	1407 (17.9)	1444 (18.4)	2851 (18.2)
χ^2^ (p-value)	–	0.59 (0.44)	–
Sexuality disclosure to heterosexual friends			
Yes	6285 (80.2)	6255 (79.8)	12,540 (80.0)
No	1556 (19.8)	1585 (20.2)	3141 (20.0)
χ^2^ (p-value)	–	0.34 (0.56)	–
Sexuality disclosure to family members			
Yes	5080 (64.8)	5159 (65.8)	10,239 (65.3)
No	2761 (35.2)	2681 (34.2)	5442 (34.7)
χ^2^ (p-value)	–	1.79 (0.18)	–
Sexuality disclosure to employers or teachers			
Yes	3237 (41.3)	3175 (40.5)	6412 (40.9)
No	4604 (58.7)	4665 (59.5)	9269 (59.1)
χ^2^ (p-value)	–	1.00 (0.32)	–
Sexuality disclosure to classmates or colleagues			
Yes	5499 (70.1)	5501 (70.2)	11,000 (70.1)
No	2342 (29.9)	2339 (29.8)	4,681 (29.9)
χ^2^ (p-value)	–	0.00 (0.96)	–
Region			
Bajío/Occidente	1426 (18.2)	1437 (18.3)	2863 (18.3)
Northwest	781 (10.0)	823 (10.5)	1604 (10.2)
South/Southeast	926 (11.8)	928 (11.8)	1854 (11.8)
CDMX/EdoMex	2662 (33.9)	2699 (34.4)	5361 (34.2)
Northeast	846 (10.8)	795 (10.1)	1641 (10.5)
Central	1200 (15.3)	1158 (14.8)	2358 (15.0)
χ^2^ (p-value)	–	3.73 (0.59)	–
Perception of community			
Tolerant of MSM			
Strongly agree	1702 (21.7)	1699 (21.7)	3401 (21.7)
Agree	2895 (36.9)	2938 (37.5)	5833 (37.2)
Neither agree nor disagree	1926 (24.6)	1892 (24.1)	3818 (24.3)
Disagree	857 (10.9)	834 (10.6)	1691 (10.8)
Strongly disagree	248 (3.2)	281 (3.6)	529 (3.4)
Missing/unknown	213 (2.7)	196 (2.5)	409 (2.6)
χ^2^ (p-value)	–	2.98 (0.56)	–
Discriminatory toward PLHIV			
Strongly agree	1298 (16.6)	1281 (16.3)	2579 (16.4)
Agree	1892 (24.1)	1963 (25.0)	3855 (24.6)
Neither agree nor disagree	1984 (25.3)	1916 (24.4)	3900 (24.9)
Disagree	1121 (14.3)	1114 (14.2)	2235 (14.3)
Strongly disagree	454 (5.8)	424 (5.4)	878 (5.6)
Missing/unknown	1092 (13.9)	1142 (14.6)	2234 (14.2)
χ^2^ (p-value)	–	3.46 (0.48)	–

EFA, exploratory factor analysis; CFA, confirmatory factor analysis; SD, standard deviation; IQR, interquartile range; HIV, human immunodeficiency virus; LGBT, lesbian, gay, bisexual, transgender; PLHIV, people living with HIV

### Item endorsement

Roughly 75% of participants (n = 11,840) endorsed ≥ 1 stigma experiences, and nearly 60% (n = 9055) endorsed ≥ 2. The most-endorsed stigma experience was verbal harassment (n = 7255; 46.3%), followed by discriminatory remarks/gossip by one’s family (n = 6450; 41.1%) and feeling scared to be in public places (n = 3756; 24.0%). The least-endorsed stigma item was sexual violence (n = 1080; 6.9%), followed by having been gossiped about by healthcare providers (n = 1217; 7.8%) and having felt mistreated in a health center (n = 1293; 8.3%).

### Exploratory and confirmatory factor analyses

Sampling adequacy was meritorious for the stigma scale (KMO = 0.85) and adequate for each individual stigma item (KMO = 0.68–0.94), indicating suitability for factor analysis. The number of eigenvalues over 1, scree plot, and results of the parallel analysis indicated a three-factor solution, which explained 66% of the variance (Additional file 1: Table S1) and exhibited good fit (Table 3). In this model, items 1–3 loaded on Factor 1, which was named “stigma from family and friends,” as the items comprising it assessed perceived exclusion from family activities, perceived discriminatory remarks/gossip by family, and perceived rejection by friends due to one’s same-sex practices. Items 4–5 loaded on Factor 2, which was named “anticipated healthcare stigma,” as the items comprising it assessed anticipatory fear and avoidance of healthcare due to worry that providers would learn about one’s same-sex practices. Items 6–13 loaded on Factor 3, which was named “general social stigma,” as the items comprising it assessed a range of negative encounters in diverse and nonspecific social contexts (e.g., gossip by healthcare providers, police refused to provide protection, verbal harassment, violence). No low loadings or cross-loadings were identified (Table 4).

**Table 3:** Fit statistics for a three-factor exploratory factor analysis in a study of sexual behavior stigma among cisgender men who have sex with men in Mexico, 2017

Factor analysis	Excluded variables	Chi-square test of model fit	RMSEA (90% CI)	CFI	TLI	SRMR
Main analysis, full sample						
Exploratory	None	621.842, df = 42, p < 0.001	0.042 (0.039, 0.045)	0.988	0.977	0.046
Confirmatory	None	1191.342, df = 62, p < 0.001	0.048 (0.046, 0.051)	0.973	0.966	0.064
Sensitivity analysis, full sample						
Exploratory	9, 10	272.446, df = 25, p < 0.001	0.036 (0.032, 0.039)	0.994	0.987	0.039
Confirmatory	9, 10	601.879, df = 41, p < 0.001	0.042 (0.039, 0.045)	0.984	0.979	0.055
Sensitivity analysis, complete cases						
Exploratory	None	448.881, df = 42, p < 0.001	0.043 (0.039, 0.046)	0.986	0.974	0.045
Confirmatory	None	934.881, df = 62, p < 0.001	0.051 (0.048, 0.054)	0.967	0.958	0.065
Exploratory	9, 10	226.345, df = 25, p < 0.001	0.039 (0.034, 0.044)	0.992	0.982	0.040
Confirmatory	9, 10	483.496, df = 41, p < 0.001	0.045 (0.041, 0.048)	0.979	0.972	0.056

RMSEA, Root Mean Square Error of Approximation; CFI, Comparative Fit Index; TLI, Tucker Lewis Index; SRMR, Standardized Root Mean Square Residual; df, degrees of freedom

Thresholds to assess fit: RMSEA < 0.05, CFI and TLI > 0.90, SRMR < 0.08

**Table 4:** Quartimin-rotated factor loadings of sexual behavior stigma items and inter-factor correlations for a three-factor model of sexual behavior stigma among cisgender men who have sex with men in Mexico, 2017

	Exploratory factor analysis (n = 7841)			Confirmatory factor analysis (n = 7840)		
	Stigma from family and friends	Anticipated healthcare stigma	General social stigma	Stigma from family and friends	Anticipated healthcare stigma	General social stigma
Factor loadings						
1. Exclusion from family activities	**0.855**	0.079	− 0.005	0.818	–	–
2. Discriminatory remarks by family	**0.778**	0.040	0.016	0.769	–	–
3. Rejection by friends	**0.470**	0.148	0.255	0.777	–	–
4. Fear of healthcare services	0.054	**0.958**	0.002	–	0.965	–
5. Avoidance of healthcare services	0.025	**0.952**	0.024	–	0.966	–
6. Felt mistreated in a health center	− 0.134	0.229	**0.725**	–	–	0.722
7. Heard providers gossiping	− 0.120	0.139	**0.774**	–	–	0.680
8. Police refusal to protect	0.074	− 0.058	**0.703**	–	–	0.675
9. Afraid to be in public places	0.275	0.128	**0.402**	–	–	0.681
10. Verbal harassment	0.342	− 0.120	**0.552**	–	–	0.761
11. Blackmail	0.180	0.041	**0.477**	–	–	0.634
12. Physical violence	0.203	− 0.181	**0.674**	–	–	0.695
13. Sexual violence	0.071	– 0.036	**0.518**	–	–	0.542
Factor correlations						
Stigma from family and friends	1.00	–	–	1.00	–	–
Anticipated healthcare stigma	0.258	1.00	–	0.445	1.00	–
General social stigma	0.563	0.404	1.00	0.766	0.490	1.00

Bolded values indicate strongest loadings ≥ 0.40

Weak to moderate inter-factor correlations were found between “stigma from family and friends” and “anticipated healthcare stigma” (r = 0.26), “stigma from family and friends” and “general social stigma” (r = 0.56), and “anticipated healthcare stigma” and “general social stigma” (r = 0.40). Internal consistency was borderline adequate for “stigma from family and friends” (α = 0.65), adequate for “anticipated healthcare stigma” (α = 0.84), and adequate for “general social stigma” (α = 0.70). In the CFA, the three-factor model demonstrated adequate fit (RMSEA = 0.048 [90% CI 0.046, 0.051]; CFI = 0.97; TLI = 0.97; SRMR = 0.06), though the RMSEA confidence interval exceeded 0.05. All items loaded above the 0.40 threshold (range: 0.54–0.97; Tables 3, 4).

### External construct validity

Greater perceived community intolerance of sexual minorities was significantly, positively associated with “stigma from family and friends” (β = 0.20; SE = 0.02; p < 0.001), “anticipated healthcare stigma” (β = 0.17; SE = 0.02; p < 0.001), and “general social stigma” (β = 0.15; SE = 0.02; p < 0.001). Lower perceived community discrimination toward PLHIV was significantly, negatively associated with “stigma from family and friends” (β = − 0.24; SE = 0.02; p < 0.001), “anticipated healthcare stigma” (β = − 0.18; SE = 0.02; p < 0.001), and “general social stigma” (β = − 0.28; SE = 0.02; p < 0.001).

### Sensitivity analyses

The EFA and CFA performed using a complete-case approach (among n = 5304 and n = 5364 participants, respectively) revealed comparable results to those of the main analysis. Three factors were indicated for extraction that explained 66% of the variance (Additional file 1: Table S1). The three-factor model exhibited good fit (Table 3), replicated the pattern of item loadings, and factor loadings and inter-factor correlations were comparable to those of the main analysis with no low loadings or cross-loadings (Additional file 1: Table S2). Internal consistency was borderline adequate for “stigma from family and friends” (α = 0.63), and adequate for “anticipated healthcare stigma” (α = 0.83), and “general social stigma” (α = 0.71). In the complete-case CFA, the three-factor structure demonstrated adequate fit (RMSEA = 0.051 [90% CI 0.048, 0.054]; CFI = 0.97; TLI = 0.96; SRMR = 0.07), akin to that of the main analysis, though the RMSEA itself exceeded 0.05. Associations between the stigma factors and both perceived community tolerance of MSM and perceived community discrimination were comparable to those in the main analysis (Table 5).

**Table 5:** Associations between stigma factors and perceived community intolerance of MSM and discrimination toward PLHIV among cisgender men who have sex with men in Mexico, available (CFA n = 7840) and complete-case (CFA n = 5364) analyses, 2017

	Stigma from family and friends			Anticipated healthcare stigma			General social stigma		
	β	SE	p-value	β	SE	p-value	β	SE	p-value
Perceived community intolerance of MSM									
Full sample	0.20	0.02	< 0.001	0.17	0.02	< 0.001	0.15	0.02	< 0.001
Complete case	0.19	0.02	< 0.001	0.16	0.02	< 0.001	0.16	0.02	< 0.001
Full sample, excluding items 9–10	0.20	0.02	< 0.001	0.17	0.02	< 0.001	0.12	0.02	< 0.001
Complete case, excluding items 9–10	0.19	0.02	< 0.001	0.16	0.02	< 0.001	0.13	0.02	< 0.001
Perceived community discrimination toward PLHIV									
Full sample	− 0.24	0.02	< 0.001	− 0.18	0.02	< 0.001	− 0.28	0.02	< 0.001
Complete case	− 0.23	0.02	< 0.001	− 0.17	0.02	< 0.001	− 0.28	0.02	< 0.001
Full sample, excluding items 9–10	− 0.22	0.02	< 0.001	− 0.17	0.02	< 0.001	− 0.28	0.02	< 0.001
Complete case, excluding items 9–10	− 0.22	0.02	< 0.001	− 0.17	0.02	0.001	− 0.31	0.02	< 0.001

MSM, men who have sex with men; PLHIV, people living with HIV; SE, standard error

Under equamax rotation, Factors 1 and 2 replicated what was found in the main analysis that used quartimin rotation. However, Factor 3 differed, as item 9 (afraid to be in public places) had subthreshold loadings on Factor 1 (0.34) as well as Factor 3 (0.34); and item 10 (verbal harassment), despite loading above the threshold on Factor 3 (0.48), cross-loaded on Factor 1 (0.43; Additional file 1: Table S3). Removing item 9 did not resolve item 10’s cross-loading, and removal of item 10 did not resolve item 9’s low loading. Both items were subsequently removed, and 3 factors remained indicated for extraction that explained 70% of the variance (Additional file 1: Table S1); no further loading issues were encountered (Additional file 1: Table S4). Weak to moderate inter-factor correlations were found between “stigma from family and friends” and “anticipated healthcare stigma” (r = 0.26), “stigma from family and friends” and “general social stigma” (r = 0.47), and “anticipated healthcare stigma” and “general social stigma” (r = 0.34). Internal consistency for “general social stigma” with items 9–10 excluded was borderline adequate (α = 0.64). The CFA of the three-factor model with items 9–10 still excluded demonstrated improved fit over previous models in which those items were included (RMSEA = 0.042 [90% CI 0.039, 0.045]; CFI = 0.98; TLI = 0.98; SRMR = 0.06), as the RMSEA and its confidence intervals were < 0.05. Associations between the stigma factors and both perceived community intolerance of MSM and perceived community discrimination toward PLHIV were comparable to those found in the previous analyses (Table 5). A complete-case approach under equamax rotation revealed EFA, CFA, and external construct validity results that were comparable to those from the previous analyses, including the loading issues encountered with items 9 and 10 (Additional file 1: Tables S5, S6, Table 5).

## Discussion

This study was undertaken to assess the factor structure, reliability, and validity of a sexual behavior stigma scale in a nationwide sample of cisgender MSM in Mexico. Three subscales emerged and were validated using confirmatory factor analysis in a separate subsample of the dataset: “stigma from family and friends,” “anticipated healthcare stigma,” and “general social stigma.” Prior research with the sexual behavior stigma items found comparable subscales, both in countries across sub-Saharan Africa, where MSM were recruited via snowball and respondent-driven sampling [53]; and in two studies in the US, where MSM were recruited online [53] and in places of social congregation [54]. Internal consistency was adequate for anticipated healthcare stigma and general social stigma but borderline adequate for stigma from family and friends. Evidence supporting external construct validity of each subscale was also established.

The “stigma from family and friends” subscale demonstrates that stigmatization by members of one’s more immediate social circle may reflect a salient, distinct domain of stigma experiences for MSM in Mexico, findings that are largely consistent with prior research, with the same (“stigma from family and friends”) or comparable subscale (“stigma from family”) emerging among a majority of MSM samples across sub-Saharan Africa and multiple samples of MSM in the US [53, 54]. All items that loaded on this subscale were intended to gauge perceived stigma, though other perceived stigma items (e.g., feeling mistreated in a health center) did not load here, indicating social context rather than stigma manifestation may be key in understanding MSM’s experiences in Mexico.

The salience of perceived stigma from family may be understood in light of socio-cultural factors such as *familismo*. Some MSM may have an unspoken agreement with their family to remain mutually silent about their sexuality, even if they have previously verbally or behaviorally (e.g., via gender nonconformity) disclosed their sexuality [20]. In such circumstances, MSM may consequently perceive any slight or potentially benign interaction negatively and as related to their sexuality [20, 80, 81]. However, it is also possible that these experiences of stigma were actually enacted by family members who were aware of participants’ sexuality (with roughly two thirds having disclosed to a family member). Indeed, experiencing stigma from family and friends/peers is common for MSM in Mexico [11, 38, 81].

“Anticipated healthcare stigma” emerged as another salient domain of stigma experienced by MSM in Mexico and was comprised of items reflecting both the same social context and same stigma type, as both items pertained to anticipatory fear and worry about healthcare workers learning of one’s same-sex practices. The emergence of these two items as a distinct subscale is consistent with all prior psychometric analyses of these stigma items across contexts [53, 54]. Notably, the one other anticipated stigma item (fear to be in public places) and other healthcare-related stigma items did not load on this subscale. “Anticipated healthcare stigma” could be tapping into participants’ tendency to refrain from disclosing their sexuality to their healthcare provider, as nondisclosure has been linked to anticipated stigma among MSM in Mexico and other contexts [4, 11, 35, 82]. However, this subscale may also be tapping into participants’ rejection sensitivities [80], as sexuality-based stigmatization by healthcare providers [35, 64] and others in one’s social network or community is not uncommon in Mexico [15, 16, 36].

Socio-structural factors such as *machismo* and fatalism already affect men’s healthcare engagement in Mexico [20, 22], which can be particularly detrimental for men at risk for or living with HIV. Environments that foster fear and avoidance of healthcare due to the possibility of stigmatization, as well as environments that foster nondisclosure of sexuality (as half of participants had not disclosed to a healthcare provider), may thwart HIV-prevention efforts even further, potentially leading to decreased uptake of HIV prevention tools (e.g., HIV testing, HIV status awareness, use of HIV pre-exposure prophylaxis) and sexual healthcare more broadly.

“General social stigma” was comprised of items that assessed stigma experiences across multiple social contexts. Most items assessed enacted stigma, but perceived and anticipated stigma items also loaded on this subscale. These findings are consistent with prior research, with some exceptions. The item pertaining to having been blackmailed loaded prominently on “general social stigma” in this study from Mexico, as it has in most sub-Saharan African country samples in which it has been assessed previously [53], but it failed to load above the 0.40 threshold in both prior US studies [53, 54]. As in sub-Saharan Africa [83], being blackmailed due to one’s same-sex practices was more commonly reported among MSM in Mexico than MSM in the US. This could suggest that country-resource level may be an important consideration in this item’s utility. The other exception pertains to the perceived and enacted healthcare stigma items, which loaded prominently on the “general social stigma” subscale in this study from Mexico and the study from the US in which MSM were recruited online [53]. In a prior study in the US with urban MSM recruited in places of social congregation [54] and in multiple prior studies with MSM recruited via snowball and respondent-driven sampling methods in sub-Saharan Africa, these items did not load as strongly [53]. Research has revealed notable sociodemographic variation among MSM samples recruited through these different methods (i.e., online vs. respondent-driven and venue-based/time-location sampling) that may drive differential stigma experiences [13, 84, 85], which may account for the observed loading differences in these healthcare stigma items. Like other stigma domains identified here, the more severe forms of stigma (i.e., discrimination, harassment, violence) comprising “general social stigma” may also be shaped by socio-structural conditions (e.g., *machismo*) and reflect the everyday realities of many MSM in Mexico [81], highlighting the need for targeted, multilevel interventions to mitigate stigma and provide recourse, support, and treatment for those affected by severe stigma.

One of our sensitivity analyses revealed less distinct subscales. Two items (fear to be in public places and verbal harassment) that strongly and distinctly loaded on “general social stigma” in the main analysis cross-loaded or weakly loaded on both “general social stigma” and “stigma from family and friends” when item complexity was permitted. This pattern of item loadings was somewhat evident in a minority of sub-Saharan African country samples in which the scale has been examined previously (e.g., Cameroon, Burkina Faso, Côte D’Ivoire) [53]. Some MSM may more openly express their sexuality in certain public spaces, and the fear they experience may be linked to the possibility of family and/or friends witnessing or learning about their sexuality through these public displays, leading to enacted stigma [17]. Verbal harassment may have cross-loaded on “stigma from family and friends” because, for some MSM, family and/or friends may have perpetrated the verbal harassment they experienced. Similarly, the item assessing experiences of having heard family members make discriminatory remarks or gossip about the participant due to their same-sex practices may have been interpreted as verbal harassment; therefore, those who endorsed the former would have also endorsed the latter. Notably, the CFA model that excluded these items demonstrated slightly better fit relative to the CFA model with these items included, though fit of both models was adequate. Additional research is needed to understand how MSM in Mexico conceive of and experience these stigmas.

External construct validity findings supported our hypotheses, with each stigma subscale exhibiting strong associations with perceived community intolerance of sexual minorities and discrimination toward PLHIV. Our hypothesis that associations with perceived community discrimination toward PLHIV would be stronger was less clearly supported, as associations between each stigma subscale and each external construct validity item were of comparable magnitude. However, absolute value comparisons showed coefficients of the associations between stigma subscales and perceived community discrimination toward PLHIV to be slightly greater than those of the associations with perceived community intolerance of sexual minorities.

### Future directions and implications

Future research with more representative samples of MSM in Mexico is needed to validate further the factor structure that we found here. Domains of stigma experiences may differ in salience for MSM residing in more rural areas, as well as for those with little or no access to the internet. Research with the subscales we identified is also needed to determine the extent to which the three stigma domains are differentially associated with HIV-related and other sexual health, mental health, and substance use outcomes. Understanding these linkages can inform the development of interventions to mitigate stigma and address its consequences. Moreover, given their brevity, these subscales could be used as brief screening tools in primary and other healthcare encounters to indicate which MSM may be at risk of experiencing a certain outcome that has been associated with the stigma domains. Finally, use of these sexual behavior stigma subscales can support public health efforts to track stigma trends, compare stigma burden across regions, support the development of additional stigma measures (including more culturally-tailored stigma measures), and document the progress of stigma mitigation interventions over time among MSM in Mexico.

### Limitations

The observed factor structure was based on stigma items that assessed lifetime experiences of stigma. Items that assess experiences of stigma within a recent or more narrowly defined time period might result in a different factor structure than what was found here. Second, though the items were intended to assess sexual behavior stigma, some participants may have responded in reference to their sexual identity or attraction instead. Stigma experiences centered on sexual identity or attraction may vary in important ways from stigma experiences centered on sexual behavior [86, 87]. Third, the items were neither created with, nor from the perspective of, MSM in Mexico and may have been less experientially or culturally relevant for participants.

Fourth, internal consistency of “stigma from family and friends” was borderline adequate. Research to construct and test additional items that may better characterize MSM’s experiences of stigma from family and friends—and therefore better correlate with other subscale items—in this setting is needed. Moreover, this subscale was comprised of only three items, and, despite high internal consistency, “anticipated healthcare stigma” was comprised of only two, potentially weakening factor reliability. Future research to identify additional experiences related to these constructs to improve each subscale’s sensitivity and discrimination between individuals who experience different levels of each type of stigma is needed. Fifth, participants were recruited via online convenience sampling, and a majority were gay-identified, < 30 years, and had a bachelor’s degree. Unstably housed MSM, those without internet access (or those who simply tend not to access the internet or use dating/hookup applications), and those who are more broadly marginalized may have been less likely to take the survey. Findings are therefore not generalizable to all MSM in Mexico. Lastly, the items used to assess external construct validity had high missingness. Moreover, only two items (rather than full-length scales) were available to assess external construct validity, and both were conceptually similar to sexual behavior stigma. Including full-length measures of constructs in the survey that are in the nomological network of, but more conceptually distinct from, sexual behavior stigma may be useful to test to provide more evidence of external construct validity.

## Conclusions

Situating the present work with earlier studies focused on these sexual behavior stigma items, findings indicate that MSM across different settings encounter sexual behavior stigma similarly. The largely congruent factor structure across regions, country-resource level, and sampling strategies adds to the evidence base supporting separate consideration of these domains of sexual behavior stigma in measurement and fills the need for improved measurement of sexual behavior stigma experienced by MSM in Mexico specifically. Moreover, that the factor structure was validated through confirmatory factor analysis and that support for construct validity was found helps to establish these subscales as valid measures of sexual behavior stigma among MSM in Mexico. Ongoing research with and utilization of these subscales can help contribute to greater understanding of sexuality-based stigma experienced by MSM in Mexico.

## Supplementary Information

**Additional file 1. **Tables S1 to S6 and Appendix.

## Data Availability

The data are not publicly available to protect confidentiality. Requests to access the data should be sent to the senior author (L. Smith).

## Abbreviations

MSM: Gay, bisexual, and other men who have sex with men
HIV: Human immunodeficiency virus
US: United States
ES: Encuesta de Sexo
CDMX/EdoMex: Mexico City
AIDS: Acquired Immune Deficiency Syndrome
PLHIV: People living with HIV
LGBT: Lesbian, gay, bisexual, transgender
KMO: Kaiser–Meyer–Olkin
EFA: Exploratory factor analysis
RMSEA: Root Mean Square Error of Approximation
CFI: Comparative Fit Index
TLI: Tucker Lewis Index
SRMR: Standardized Root Mean Square Residual
CFA: Confirmatory factor analysis
