# Supplementary material for: Metrics of sexual behavior stigma among cisgender men who have sex with men in Mexico: exploratory and confirmatory factor analyses
Source: BMC Infect Dis. 2022 Aug 13;22:690. doi: 10.1186/s12879-022-07672-0 (PMC9375942; doi:10.1186/s12879-022-07672-0)
Supplement: Supplementary file 1 — Additional file 1. Tables S1 to S6 and Appendix. [file 12879_2022_7672_MOESM1_ESM.docx]

| Table S1. Parallel analyses of sexual behavior stigma items administered to cisgender men who have sex with men in Mexico, 2017. | | | |
| --- | --- | --- | --- |
| Sample (items excluded) | Parallel analyses | Number of factors to extract | Number of components (% variance explained) |
| Full (none)  (N=7,841) | 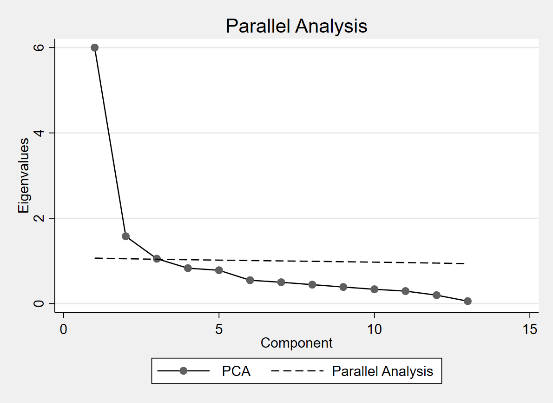 | 2-3 | 2 (58%), 3 (66%) |
| Full (9, 10)  (N=7,841) | 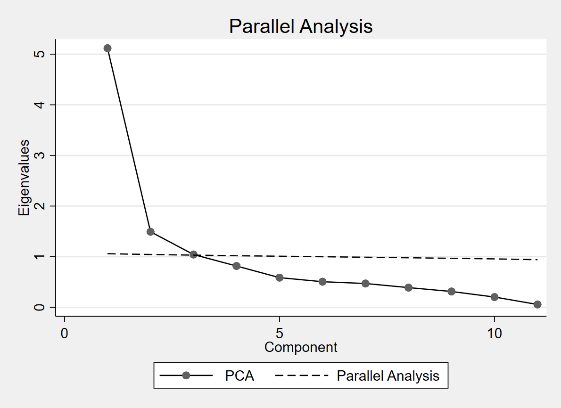 | 2-3 | 2 (60%), 3 (70%) |
| Complete case (none)  (N=5,304) | 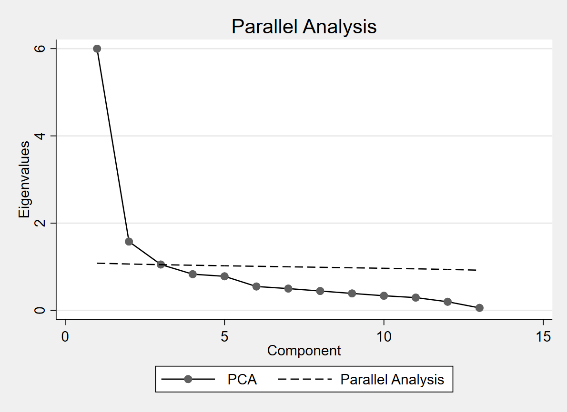 | 2-3 | 2 (58%), 3 (66%) |
| Complete case (9, 10)  (N=5,304) | 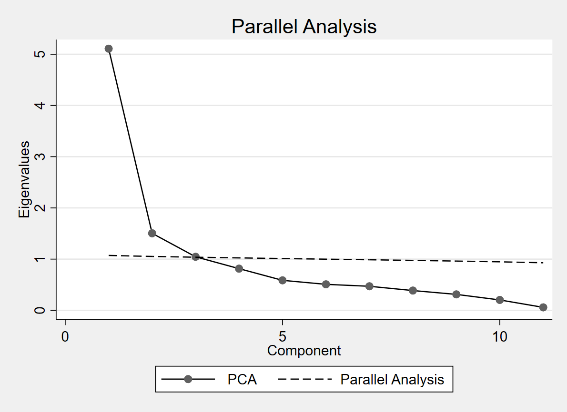 | 2-3 | 2 (60%), 3 (70%) |

| Table S2. Quartimin-rotated factor loadings of sexual behavior stigma items and inter-factor correlations for a three-factor model of sexual behavior stigma among cisgender men who have sex with men in Mexico, complete-case analysis, 2017. | | | | | | |
| --- | --- | --- | --- | --- | --- | --- |
|  | Exploratory factor analysis (n=5,304) | | | Confirmatory factor analysis (n=5,364) | | |
|  | Stigma from family and friends | Anticipated healthcare stigma | General  social  stigma | Stigma from family and friends | Anticipated healthcare stigma | General  social  stigma |
| Factor loadings |  |  |  |  |  |  |
| 1. Exclusion from family activities | **0.866** | 0.057 | -0.014 | **0.813** | - | - |
| 2. Discriminatory remarks by family | **0.769** | 0.026 | 0.044 | **0.770** | - | - |
| 3. Rejection by friends | **0.447** | 0.159 | 0.258 | **0.781** | - | - |
| 4. Fear of healthcare services | 0.035 | **0.961** | -0.001 | - | **0.965** | - |
| 5. Avoidance of healthcare services | 0.024 | **0.952** | 0.021 | - | **0.959** | - |
| 6. Felt mistreated in a health center | -0.118 | 0.200 | **0.712** | **-** | **-** | **0.712** |
| 7. Heard providers gossiping | -0.124 | 0.136 | **0.781** | **-** | **-** | **0.686** |
| 8. Police refusal to protect | 0.023 | -0.065 | **0.753** | **-** | **-** | **0.662** |
| 9. Afraid to be in public places | 0.229 | 0.110 | **0.460** | **-** | **-** | **0.685** |
| 10. Verbal harassment | 0.295 | -0.101 | **0.578** | **-** | **-** | **0.777** |
| 11. Blackmail | 0.120 | 0.079 | **0.526** | **-** | **-** | **0.665** |
| 12. Physical violence | 0.179 | -0.163 | **0.691** | **-** | **-** | **0.712** |
| 13. Sexual violence | 0.072 | -0.031 | **0.529** | **-** | **-** | **0.557** |
| Factor correlations |  |  |  |  |  |  |
| Stigma from family and friends | 1.00 | - | - | 1.00 | - | - |
| Anticipated healthcare stigma | 0.249 | 1.00 | - | 0.423 | 1.00 | - |
| General social stigma | 0.585 | 0.422 | 1.00 | 0.767 | 0.482 | 1.00 |

Bolded values indicate strongest loadings ≥0.40

| Table S3. Equamax-rotated factor loadings of sexual behavior stigma items and inter-factor correlations for a three-factor model of sexual behavior stigma among cisgender men who have sex with men in Mexico, 2017. | | | |
| --- | --- | --- | --- |
|  | Exploratory factor analysis (n=7,841) | | |
|  | Stigma from family and friends | Anticipated healthcare stigma | General social  stigma |
| Factor loadings |  |  |  |
| 1. Exclusion from family activities | **0.869** | 0.086 | -0.044 |
| 2. Discriminatory remarks by family | **0.795** | 0.047 | -0.022 |
| 3. Rejection by friends | **0.513** | 0.180 | 0.203 |
| 4. Fear of healthcare services | 0.046 | **0.964** | -0.012 |
| 5. Avoidance of healthcare services | 0.020 | **0.960** | 0.008 |
| 6. Felt mistreated in a health center | -0.038 | 0.307 | **0.646** |
| 7. Heard providers gossiping | -0.016 | 0.222 | **0.689** |
| 8. Police refusal to protect | 0.174 | 0.017 | **0.620** |
| 9. Afraid to be in public places | 0.335 | 0.174 | 0.342 |
| 10. Verbal harassment | 0.426 | -0.059 | **0.475** |
| 11. Blackmail | 0.250 | 0.094 | **0.414** |
| 12. Physical violence | 0.302 | -0.109 | **0.590** |
| 13. Sexual violence | 0.145 | 0.020 | **0.456** |
| Factor correlations |  |  |  |
| Stigma from family and friends | 1.00 | - | - |
| Anticipated healthcare stigma | 0.273 | 1.00 | - |
| General social stigma | 0.479 | 0.310 | 1.00 |

Bolded values indicate strongest loadings ≥0.40; shaded cells indicate low loadings or cross-loadings

| Table S4. Equamax-rotated factor loadings of sexual behavior stigma items and inter-factor correlations for a three-factor model of sexual behavior stigma among cisgender men who have sex with men in Mexico, 2017. | | | | | | |
| --- | --- | --- | --- | --- | --- | --- |
|  | Exploratory factor analysis (n=7,841) | | | Confirmatory factor analysis (n=7,840) | | |
|  | Stigma from family and friends | Anticipated healthcare stigma | General social  stigma | Stigma from family and friends | Anticipated healthcare stigma | General social  stigma |
| Factor loadings |  |  |  |  |  |  |
| 1. Exclusion from family activities | **0.869** | 0.044 | -0.032 | **0.830** | - | - |
| 2. Discriminatory remarks by family | **0.808** | 0.016 | 0.012 | **0.771** | - | - |
| 3. Rejection by friends | **0.505** | 0.179 | 0.207 | **0.762** | - | - |
| 4. Fear of healthcare services | 0.030 | **0.951** | 0.004 | - | **0.955** | - |
| 5. Avoidance of healthcare services | 0.020 | **0.965** | 0.019 | - | **0.977** | - |
| 6. Felt mistreated in a health center | 0.026 | 0.227 | **0.664** | **-** | **-** | **0.785** |
| 7. Heard providers gossiping | 0.031 | 0.158 | **0.735** | **-** | **-** | **0.737** |
| 8. Police refusal to protect | 0.172 | -0.011 | **0.628** | **-** | **-** | **0.661** |
| 9. Afraid to be in public places^a^ | **-** | **-** | **-** | **-** | **-** | **-** |
| 10. Verbal harassment^a^ | **-** | **-** | **-** | **-** | **-** | **-** |
| 11. Blackmail | 0.223 | 0.126 | **0.445** | **-** | **-** | **0.653** |
| 12. Physical violence | 0.311 | -0.076 | **0.543** | **-** | **-** | **0.650** |
| 13. Sexual violence | 0.181 | 0.006 | **0.465** | **-** | **-** | **0.540** |
| Factor correlations |  |  |  |  |  |  |
| Stigma from family and friends | 1.00 | - | - | 1.00 | - | - |
| Anticipated healthcare stigma | 0.263 | 1.00 | - | 0.445 | 1.00 | - |
| General social stigma | 0.472 | 0.335 | 1.00 | 0.735 | 0.496 | 1.00 |

Bolded values indicate strongest loadings ≥0.40

^a^Excluded

| Table S5. Equamax-rotated factor loadings of sexual behavior stigma items and inter-factor correlations for a three-factor model of sexual behavior stigma among cisgender men who have sex with men in Mexico, complete-case analysis, 2017. | | | |
| --- | --- | --- | --- |
|  | Exploratory factor analysis (n=5,304) | | |
|  | Stigma from family and friends | Anticipated healthcare stigma | General social  stigma |
| Factor loadings |  |  |  |
| 1. Exclusion from family activities | **0.885** | 0.057 | -0.060 |
| 2. Discriminatory remarks by family | **0.795** | 0.032 | -0.004 |
| 3. Rejection by friends | **0.500** | 0.191 | 0.196 |
| 4. Fear of healthcare services | 0.027 | **0.968** | -0.018 |
| 5. Avoidance of healthcare services | 0.020 | **0.961** | 0.001 |
| 6. Felt mistreated in a health center | -0.005 | 0.284 | **0.617** |
| 7. Heard providers gossiping | 0.000 | 0.228 | **0.678** |
| 8. Police refusal to protect | 0.149 | 0.022 | **0.648** |
| 9. Afraid to be in public places | 0.309 | 0.165 | 0.382 |
| 10. Verbal harassment | 0.399 | -0.034 | **0.484** |
| 11. Blackmail | 0.210 | 0.141 | **0.445** |
| 12. Physical violence | 0.299 | -0.083 | **0.588** |
| 13. Sexual violence | 0.162 | 0.030 | **0.452** |
| Factor correlations |  |  |  |
| Stigma from family and friends | 1.00 | - | - |
| Anticipated healthcare stigma | 0.274 | 1.00 | - |
| General social stigma | 0.493 | 0.323 | 1.00 |

Bolded values indicate strongest loadings ≥0.40; shaded cells indicate low loadings or cross-loadings

| Table S6. Equamax-rotated factor loadings of sexual behavior stigma items and inter-factor correlations for a three-factor model of sexual behavior stigma among cisgender men who have sex with men in Mexico, complete-case analysis, 2017. | | | | | | |
| --- | --- | --- | --- | --- | --- | --- |
|  | Exploratory factor analysis (n=5,304) | | | Confirmatory factor analysis (n=5,364) | | |
|  | Stigma from family and friends | Anticipated healthcare stigma | General  social  stigma | Stigma from family and friends | Anticipated healthcare stigma | General  social  stigma |
| Factor loadings |  |  |  |  |  |  |
| 1. Exclusion from family activities | **0.869** | 0.044 | -0.032 | **0.824** | - | - |
| 2. Discriminatory remarks by family | **0.808** | 0.016 | 0.012 | **0.771** | - | - |
| 3. Rejection by friends | **0.505** | 0.179 | 0.207 | **0.767** | - | - |
| 4. Fear of healthcare services | 0.030 | **0.951** | 0.004 | - | **0.956** | - |
| 5. Avoidance of healthcare services | 0.020 | **0.965** | 0.019 | - | **0.968** | - |
| 6. Felt mistreated in a health center | 0.026 | 0.227 | **0.664** | **-** | **-** | **0.775** |
| 7. Heard providers gossiping | 0.031 | 0.158 | **0.735** | **-** | **-** | **0.740** |
| 8. Police refusal to protect | 0.172 | -0.011 | **0.628** | **-** | **-** | **0.651** |
| 9. Afraid to be in public places^a^ | **-** | **-** | **-** | **-** | **-** | **-** |
| 10. Verbal harassment^a^ | **-** | **-** | **-** | **-** | **-** | **-** |
| 11. Blackmail | 0.223 | 0.126 | **0.445** | **-** | **-** | **0.677** |
| 12. Physical violence | 0.311 | -0.076 | **0.543** | **-** | **-** | **0.658** |
| 13. Sexual violence | 0.181 | 0.006 | **0.465** | **-** | **-** | **0.545** |
| Factor correlations |  |  |  |  |  |  |
| Stigma from family and friends | 1.00 | - | - | 1.00 | - | - |
| Anticipated healthcare stigma | 0.263 | 1.00 | - | 0.422 | 1.00 | - |
| General social stigma | 0.472 | 0.335 | 1.00 | 0.744 | 0.502 | 1.00 |

Bolded values indicate strongest loadings ≥0.40

^a^Excluded

Appendix

Spanish version of sexual behavior stigma items, as well as the instructions on responding to the items, presented to participants taking ES Entres Hombres (English version follows):

Las siguientes preguntas se tratan de cosas que puedes haber experimentado alguna vez porque alguien sabía o suponía que tienes relaciones sexuales con hombres.

1. ¿Alguna vez te has sentido excluido de actividades familiares porque tienes relaciones sexuales con hombres?
2. ¿Alguna vez has sentido que miembros de tu familia hayan hecho comentarios discriminatorios o chismes acerca de ti porque tienes relaciones sexuales con hombres?
3. ¿Alguna vez te has sentido rechazado por tus amigos porque tienes relaciones sexuales con hombres?
4. ¿Alguna vez has sentido miedo de acudir a servicios médicos porque te preocupa que alguien pueda darse cuenta de que tienes relaciones sexuales con hombres?
5. ¿Alguna vez has evitado acudir a servicios médicos porque te preocupa que alguien pueda darse cuenta que tienes relaciones sexuales con hombres?
6. ¿Alguna vez has sentido que no te trataron bien en un centro de salud porque alguien sabía que tienes relaciones sexuales con hombres?
7. ¿Alguna vez has escuchado a los proveedores de salud hacer comentarios discriminatorios o chismes acerca de ti (hablar de ti) porque tienes relaciones sexuales con hombres?
8. ¿Alguna vez has sentido que la policía se negó a protegerte porque tienes relaciones sexuales con hombres?
9. ¿Alguna vez has sentido miedo de estar en lugares públicos porque tienes relaciones sexuales con hombres?
10. ¿Alguna vez has sido verbalmente acosado y pensaste que era porque tienes relaciones sexuales con hombres?
11. ¿Alguna vez has sido chantajeado por alguien porque tienes sexo con hombres?
12. ¿Alguna vez has sido físicamente lastimado (empujado, zarandeado, abofeteado, golpeado, pateado, estrangulado o herido físicamente de alguna manera)?
    1. ¿Consideras que alguna de estas experiencias de violencia física fue/o fueron relacionadas con el hecho de que tienes sexo con hombres?
13. ¿Alguna vez has sido obligado a tener relaciones sexuales cuando usted no querías hacerlo? (Por forzado, quiero decir físicamente forzado, coaccionado a tener relaciones sexuales o penetrado con un objeto, cuando tú no querías).
    1. ¿Consideras que cualquiera de estas experiencias de violencia sexual se relaciona con el hecho de que tienes sexo con hombres?

Response options in Spanish for each item

Si No No aplica Prefiero no contestar No sé

The following questions are about things you may have experienced because someone knew or assumed you have sex with men.

1. Have you ever felt excluded from family activities because you have sex with men?
2. Have you ever felt that members of your family have made discriminatory comments or gossiped about you because you have sex with men?
3. Have you ever felt rejected by your friends because you have sex with men?
4. Have you ever been afraid to go to medical services because you were worried that someone might realize that you have sex with men?
5. Have you ever avoided going to medical services because you were worried that someone might realize you have sex with men?
6. Have you ever felt like you weren't treated well at a health center because someone knew you have sex with men?
7. Have you ever heard health care providers make discriminatory comments or gossip about you (talking about you) because you have sex with men?
8. Have you ever felt that the police refused to protect you because you have sex with men?
9. Have you ever felt afraid to be in public places because you have sex with men?
10. Have you ever been verbally harassed and thought it was because you have sex with men?
11. Have you ever been blackmailed by someone because you have sex with men?
12. Have you ever been physically hurt (pushed, shaken, slapped, beaten, kicked, strangled, or physically injured in any way)?
    1. Do you believe that any of these experiences of physical violence were related to the fact that you have sex with men?
13. Have you ever been forced to have sex when you didn't want to? (By forced, I mean physically forced, coerced into having sex, or penetrated with an object, when you didn't want to.)
    1. Do you believe that any of these experiences of sexual violence were related to the fact that you have sex with men?

Response options in for each item

Yes No Not applicable Prefer not to answer I don’t know
